# Exploring the use of mobile translation applications for culturally and linguistically diverse patients during medical imaging examinations in Australia – a systematic review

**DOI:** 10.1002/jmrs.755

**Published:** 2024-01-22

**Authors:** Bridget Taylor, Glenda McLean

**Affiliations:** ^1^ Monash Imaging, Monash Health Berwick Victoria Australia

**Keywords:** Communication, culturally and linguistically diverse, healthcare, mobile translation applications, radiology

## Abstract

Australian healthcare provides services to a vast culturally and linguistically diverse (CALD) population. Professional interpreters are the gold standard for medical interpretation during healthcare interactions with CALD patients with limited English proficiency (LEP). However, accessing interpretation services can be difficult and may not be appropriate when timely translation is needed. Mobile translation applications (MTAs) have been suggested as a way for healthcare workers (HCWs) to provide timely translation when engaging with CALD patients. This systematic review aimed to investigate the potential for MTAs to be used in Australian medical imaging (MI) departments to enhance communication and safety for CALD patients and HCWs. Enablers and limitations of MTAs were appraised for use in MI and important design considerations suggested. Results found that MTAs may enhance communication between CALD patients and MI professionals and uphold safety by more accurately performing procedure matching and healthcare assessments. MTAs also offer readily available translation during out of hours care, emergency scenarios and everyday care. However, reliability of free‐input translation and patient confidentiality were flagged as important limitations of MTAs that need to be addressed should a safe MTA be designed for MI purposes. MTAs also need to be designed with consideration for CALD patients who have low literacy levels and mental impairment. Devices should be installed close to the point of care to enable MI professionals to easily retrieve and use the MTA. MTAs used in this way can potentially improve care of CALD patients in MI when professional interpreters are absent.

## Graphical abstract

In this review, we sought to investigate the potential for mobile translation applications (MTAs) to be used during medical imaging examinations of culturally and linguistically diverse (CALD) patients. We found that MTAs may enhance communication between CALD patients and medical imaging professionals and uphold safety by more accurately performing procedure matching and healthcare assessments. Importantly, reliability of free‐input translation and patient confidentiality were flagged as limitations of MTAs that need to be addressed should a safe MTA be designed for medical imaging purposes.

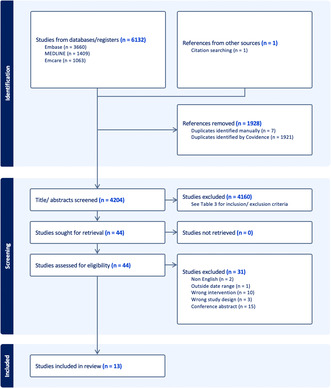

## Introduction

Australian healthcare provides services to a vast culturally and linguistically diverse (CALD) population. Australian census results indicated that over 7 million people (27.6%) living in Australia in 2021 were born overseas.^1^ Furthermore, the percentage of Australians speaking a language other than English at home has risen from 21% in 2016 to 22.3% in 2021.^2^, ^3^ Importantly, 18% of Australians speaking another language do not speak English well or at all.^2^ With such a diverse population, it is imperative that all patients in Australia have access to safe and effective healthcare that is culturally and linguistically appropriate.

Use of professional interpreters is recommended for interactions with CALD patients who have low English proficiency (LEP) during a healthcare interaction to reduce the risk of miscommunication between patient and the healthcare worker (HCW).^4^, ^5^ Professional interpreters are highly skilled in converting oral phrases from one language to another to preserve meaning and uphold professional ethics in a culturally sensitive way during a healthcare interaction.^4^, ^5^ For this reason, they have been described as the ‘gold standard’ for medical translation.^4^ Engaging a professional interpreter may not always be feasible, however, for quick, low‐risk interactions or in emergency situations. The Australian Department of Health and Human Services (DHHS) language services policy recommends delaying the examination/ appointment where appropriate, or in emergency situations, engage with an interpreter that is accredited at a lower level.^5^ Bi‐lingual staff or ad hoc interpreters are only recommended to make translations for low‐risk conversations, and are prone to making translation errors due to personal bias or sub‐par language skills.^5^, ^6^

In the context of medical imaging (MI), quick, lower‐risk interactions by radiographers, sonographers and nuclear medicine technologists with patients include confirming patient identity, anatomical site/side of concern, pregnancy status and giving positioning instructions. Indeed, procedure matching and confirming 3C's (correct patient, correct procedure, correct site & side) was identified as an integral way to reduce radiation safety incidents in Australia, of which 65% were caused by human error in 2020.^7^ Higher‐risk conversations may include completing the iodinated contrast safety questionnaire, magnetic resonance imaging (MRI) safety screening or gaining consent for invasive imaging (e.g. transvaginal ultrasound). It is not disputed that a professional interpreter is needed for such high‐risk conversations as miscommunication may result in poor outcomes.^4^

Mobile translation applications (MTAs) have been posed as a solution for CALD patients and their HCWs to access timely translation for low‐risk conversations in the absence of professional interpreters.^6^, ^8^, ^9^, ^10^, ^11^, ^12^ They may also provide an impartial translation compared to ad hoc interpreters, where personal language competency, cultural values and relation to the patient may result in a biased or incomplete translation.^4^ MTAs vary depending on their mode of translation, including free input text‐to‐text, speech‐to‐speech or pre‐loaded phrases.^10^, ^13^ Audiovisual elements may also aid the translation.^13^, ^14^ Currently, there are examples of MTAs with pre‐loaded phrases in use by major Victorian public hospitals, such as CALD Assist™ and TalkToMe™. These applications have been developed for use by medical, nursing and allied health staff for low‐risk, quick translations where engaging with a professional interpreter would be unnecessary. Free‐text and free‐speech input MTAs, notably Google Translate™, have also been investigated for use in healthcare.^10^, ^11^, ^13^, ^15^, ^16^ Additionally, pre‐loaded phrases have been included on CT scanners in the form of multi‐language voice recordings to increase image quality and reduce motion artefact.^17^

Although the use of MTAs has been explored in a variety of healthcare disciplines, there is very limited literature surrounding the technology's use in MI settings. Hence, this paper aims to investigate how MTAs may be designed and used in MI departments to enhance communication and safety for CALD patients and HCWs in the absence of a professional interpreter. We performed a systematic review of global literature surrounding the use of MTAs across the healthcare disciplines in order to theoretically apply the literature to the Australian MI setting and discuss benefits and limitations of the technology relating to how MTAs can enhance the experiences of CALD patients during MI examinations.

## Methods

### Search strategy

Prior to performing a literature search, the research question was formulated using the PICO framework (Table 1). The PRISMA 2020 checklist was completed to ensure that the rationale, hypothesis and intended methods for the review were defined (see Appendix S1).^18^

**Table 1:** PICO framework used to develop research question.

Population	CALD MI patients in Australia
Intervention	MTAs
Control/Comparison	Professional Interpreters
Outcome	MTA designed and used to be effective in enhancing safety and understanding of MI examinations for CALD patients

CALD, culturally and linguistically diverse; MI, medical imaging; MTA(s), mobile translation application(s).

The search strategy was created by expanding synonyms for keywords from the PICO framework and manually searching for related MeSH terms (Table 2). A preliminary Google Scholar search found that there was very limited literature reporting on the use of MTAs in MI settings. Hence, the population was expanded in the search strategy to include MTAs more broadly used in healthcare settings. The search strategy was applied to three databases: Ovid Medline, Embase and Emcare (Table 2). These databases were chosen as they are three prominent databases for biomedical research.^19^, ^20^, ^21^ The search period was filtered to 2012 to 2023 and no filters were applied to the language of the paper. Citation searching was conducted after full‐text screening to include potential articles missed in the database search.

**Table 2:** Search terms applied during database searching.

Line #	Search terms	Ovid Medline	Embase	Ovid Emcare
Line 1	Limited English Proficiency/ or Communication Barriers/ or Multilingualism/ [MeSH terms]	8099469	5967	1170
Line 2	((culturally and linguistically diverse) or CALD or non‐English speak* or diverse communities or healthcare or patient*).ti,ab. [keywords]	8097449	12029256	2651330
Line 3	Mobile Applications/ or Smartphone/ or Cell Phone/ or Audiovisual Aids/ or Translating/ [MeSH terms]	38900	60510	15020
Line 4	(Translation app* or mobile translation app* or digital translator or iPad or tablet or Android or iPhone or translat*).ti,ab. [keywords]	426474	562550	103577
Line 5	(Safety or understand* or communicat*).ti,ab. [keywords]	2446646	3279647	867709
Line 6	Health Literacy/ or Comprehension/	25086	52772	19821
Line 7	Health Personnel/ or (Radiology/ or Radiology Department, Hospital/) or Hospitals/ or Radiograph*/ [MeSH terms]	188632	516953	198191
Line 8	(Allied health* or hospital or medical imaging).ti,ab. [keywords]	1228210	1959452	497091
Line 9	Line 1 or 2	8108107	12032549	2651878
Line 10	Line 3 or 4	459387	615957	117185
Line 11	Line 5 or 6	2460908	3310461	879803
Line 12	Line 7 or 8	1351345	2284263	597448
Line 13	Line 9 and 10 and 11 and 12	2534	6434	1641
Line 14	Limit 13 to yr = ‘2012 to current’	2074	5469	1278
Line 15	Limit 14 to abstracts	2069	5458	1278
Line 16	Line 7 or 15	190150	520515	199066
Line 17	Line 13 and 16	2154	6024	1526
Line 18	(Professional interpreter or face‐to‐face interpreter or language interpreter or interpreting service* or professional translat* or translat*).ti,ab.	390942	500170	93110
Line 19	Line 17 and 18	1405	3660	1063

CALD, culturally and linguistically diverse.

### Data collection

Proceeding the literature search, the resultant articles were entered into Covidence (Covidence systematic review software, Veritas Health Innovation, Melbourne, Australia. Available at www.covidence.org) where duplicates were removed. Inclusion and exclusion criteria (Table 3) were written and applied independently and blindly by two reviewers (B.Taylor and G.McLean) during initial title and abstract screening of all articles. The remaining articles were sourced for full‐text review and the inclusion and exclusion criteria were again applied independently and blindly by the two reviewers. Any discrepancies between the reviewers during article screening were verbally discussed and resolved. One reviewer extracted data from each article relating to aims, method, key findings, and translation mode. Data were presented in tabular form (Table 4).

**Table 3:** Inclusion/exclusion criteria.

	Inclusion	Exclusion
Article type	Published from 2012 to 2023 Original article, pilot study	Published prior to 2012 Systematic review, literature review, case study, conference proceedings abstract, editorial, opinion piece, book chapter, study protocol
Participants	CALD patients who receive care in a language they are not proficient in HCWs who are required to deliver care to CALD patients in a language the patient is not proficient in Adult CALD patients receiving care from medical, allied health or nursing professionals	Patients who receive care in a language they are proficient in HCWs who are required to deliver care to patients in a language the patient is proficient in Patients whose language barrier is not the result of cultural and linguistic diversity (eg. Hearing/vision impaired, disability) Paediatric patients
Intervention	Mobile technology/ applications/ websites that facilitate a language translation in these formats: free‐input speech‐to‐speech, speech‐to‐text, text‐to‐speech, text‐to‐text translation or pre‐loaded phrases embedded images or videos MTA used during a healthcare interaction Professional interpreters used as the control/gold standard	technology that does not facilitate a language translation technology not used to facilitate healthcare interaction translation service not accessible on a computer, mobile or portable device
Outcomes	Outcomes relating to safety and understanding for CALD patients and their HCW during healthcare interactions Measured or self‐reported Outcomes relating to acceptance and satisfaction with MTAs by CALD patients and their HCW Outcomes relating to important features/phrases for creation of a healthcare appropriate MTA	

CALD, culturally and linguistically diverse; HCW(s), healthcare worker(s); MTA(s), mobile translation applications.

**Table 4:** Summary of 13 articles included in the review.

Article	CASP rating %	Translation mode	Country of origin & native language	Study aim	Participants & setting	Method	Results
Albrecht et al. 2013^8^	95	XPrompt app	Germany German	Explore staff experiences in hospital wards after using mobile translation app with CALD pt's	*n* = 42 nursing staff 10 public and private wards, 1 Hospital	Likert scale survey Nurses rating experience with XPrompt after 6 weeks of use	Useful for daily interactions User‐friendly app for nurses Elderly pt's have difficulty with app use
Choong et al. 2021^24^	70	Audio recordings on laptop	Singapore English	Investigate usefulness of audio recordings to communicate instructions to CALD pt's during CXR	*n* = 22 radiographers 1 general X‐ray department	Likert scale survey of radiographers using recordings Reject rate analysis of CXR's	49% had trouble having pt follow breathing instructions with recordings 54.5% suggest they can achieve CXR in full inspiration with recordings 95.5% suggested it was useful
Davis et al. 2019^28^	100	Professional interpreters, bilingual staff, Google translate, Medical translation software	America English	Investigate difficulties relating to translating pt discharge instructions and suggest strategies for improvement	*n* = 31 American hospitals with paediatric inpatients	Online survey Online environmental scan of hospital language service policy documents	Most instructions pre‐translated (87%) or by professional interpreters (81%) Machine translation alone is forbidden in 5 hospitals
Day & Song 2017^6^	95	Listen Please app	New Zealand English	Evaluate translation app ‘Listen Please’ and evaluate usefulness in clinical setting	*n* = 15 clinical staff (doctors & nurses) One hospital	Technology Acceptance Model 2 Framework used for focus group interviews Clinicians asked to use app as part of a simulation Thematic analysis	App was easy to learn (100%) and use (93%) App may be useful for clinician's patients (93%) Concern about digital literacy of older patients and colleagues
Freyne et al. 2018^9^	100	CALD assist app	Australia English	To design and appraise CALD assist app	2 Victorian Hospitals	45 allied health clinicians User needs analysis from focus group interviews	Time taken to do health assessment reduced to 15.6 min from 42 min Confidence in quality of health assessment rose to 42%
Freyne et al. 2015^14^	55	CALD assist app	Australia English	Report progress of app development, specifically needs of allied health for the app	*n* = 19 allied health clinicians Victorian public hospital	Focus group interviews User needs analysis	Introduction/dismissal flagged as very important phrases Pictures/videos important inclusions Suggested that limited health assessments can be done with app
Hwang et al. 2022^10^	100	Google Translate, CALD assist app, Talk To Me app	Australia English	Trial MTAs to enhance patient/clinician communication	*n* = 4 Aged care wards Australian hospitals	2 month trial of apps Interactions with app recorded and analysed Thematic analysis	Staff agreed apps were useful and would use again (65%) Frustration and confusion experienced with Google Translate Pt's experienced frustration replying with CALD Assist & Talk to Me due to one‐directional design
Miller et al. 2018^16^	75	Google Translate	America English	Explore if Google Translate is safe to translate PCI's	*n* = 100 PCI's provided for translation 6 English/Spanish translators & 6 English HCW's	Input PCI's into Google Translate to Spanish & back‐translate into English Panel evaluated Spanish translation and English back translation with Likert scales	9 English to Spanish translations were ‘unsafe’ More complex instructions are prone to incorrect translation Good English to Spanish translation did not assure good back translation to English
Panayiotou et al. 2020^11^	95	CALD Assist, TalkToMe, Google Translate	Australia English	Explore perceptions of older CALD pt's and HCW's towards MTAs in HC interactions	12 older CALD pt's 17 HCW's 3 Australian public hospitals	Focus group interviews with CALD pt's & HCW's Inductive content analysis Survey rating MTAs	MTAs can reduce communication barriers Experienced further confusion translating complex medical reports Helps to build rapport Digital literacy is a barrier
Panayiotou et al. 2019^11^	95	15 iPad compatible MTAs	Australia English	Review iPad compatible translation apps for everyday healthcare applications	*n* = 15 MTAs iPad compatible	Search for free iPad apps Feature analysis Analysis of everyday HC use	6/15 specifically for HC 2 apps only capable to 2‐way conversation Essentials: low cost, able to be used offline, no ads, able to be used on lots of platforms Policy, data security, confidentiality all important considerations
Pocock et al. 2020^15^	100	n/a	Malaysia & Thailand Malay; Thai	Investigate stakeholder views on migrants' and HCW's language competency	*n* = 44 Malaysian industry stakeholders and HCW's *n* = 50 Thai industry stakeholders and HCW's	Informal discussions with stakeholders Meta‐analysis of interviews & policy documents	Describe language barriers for migrant workers as a ‘problem’ Bedside manner from doctors to migrants is poor Difficulty obtaining informed consent with migrants Google Translate suggested as way to overcome language barrier
Silvera‐Tawil et al. 2021^12^	84	CALD assist app	Australia English	Evaluate use of CALD assist in nursing of CALD patients	4 inpatient wards over 3 Australian hospitals *n* = 30 staff surveyed *n* = 7 pt's surveyed	Baseline observations & staff survey Record interactions with app and observations, pt interviews Posttrial staff focus group & survey	Staff less frustrated communicating with CALD pt's using the app (*P* = 0.008) 86% of pt's indicated app assisted them understanding Need for having iPads with app close to point of care Suggested as useful in identifying pain
van Vuuren, van Dyk & Mokoena 2021^25^	85	n/a	South Africa Afrikaans, English, Xhosa, Zulu, Southern Sotho, Northern Sotho, Twana, Tsonga, Venda, Swati, Southern Africal Sign, Ndebele	Investigate experiences of radiographers communicating with CALD pt's in South Africa	*n* = 18 South African radiographers One radiology department	Focus group interviews	Suggestion for posters and visual aids to translate key phrases. Also useful for deaf patients MTAs suggested when translators are absent. May not be good for all languages/dialects

CALD, culturally and linguistically diverse; CXR, chest X‐ray; HC, healthcare; HCW, healthcare worker; MTA, mobile translation application; PCI, patient care instructions; pt, patient.

### Risk of bias assessment

The CASP checklist was used independently and blindly by the two reviewers to conduct a risk of bias assessment of the articles included for review (see Appendix S2).^22^, ^23^ This checklist was chosen as the screening questions were appropriate for the qualitative style of articles that were included in the review. In this tool, articles were scored against the checklist questions with 2 indicating yes, 1 indicating unclear, 0 indicating no. Total scores were represented as a percentage, with a higher percentage indicated less risk of bias. Where there was a discrepancy, the reviewers reached consensus through discussion.

## Results

**Figure 1:**
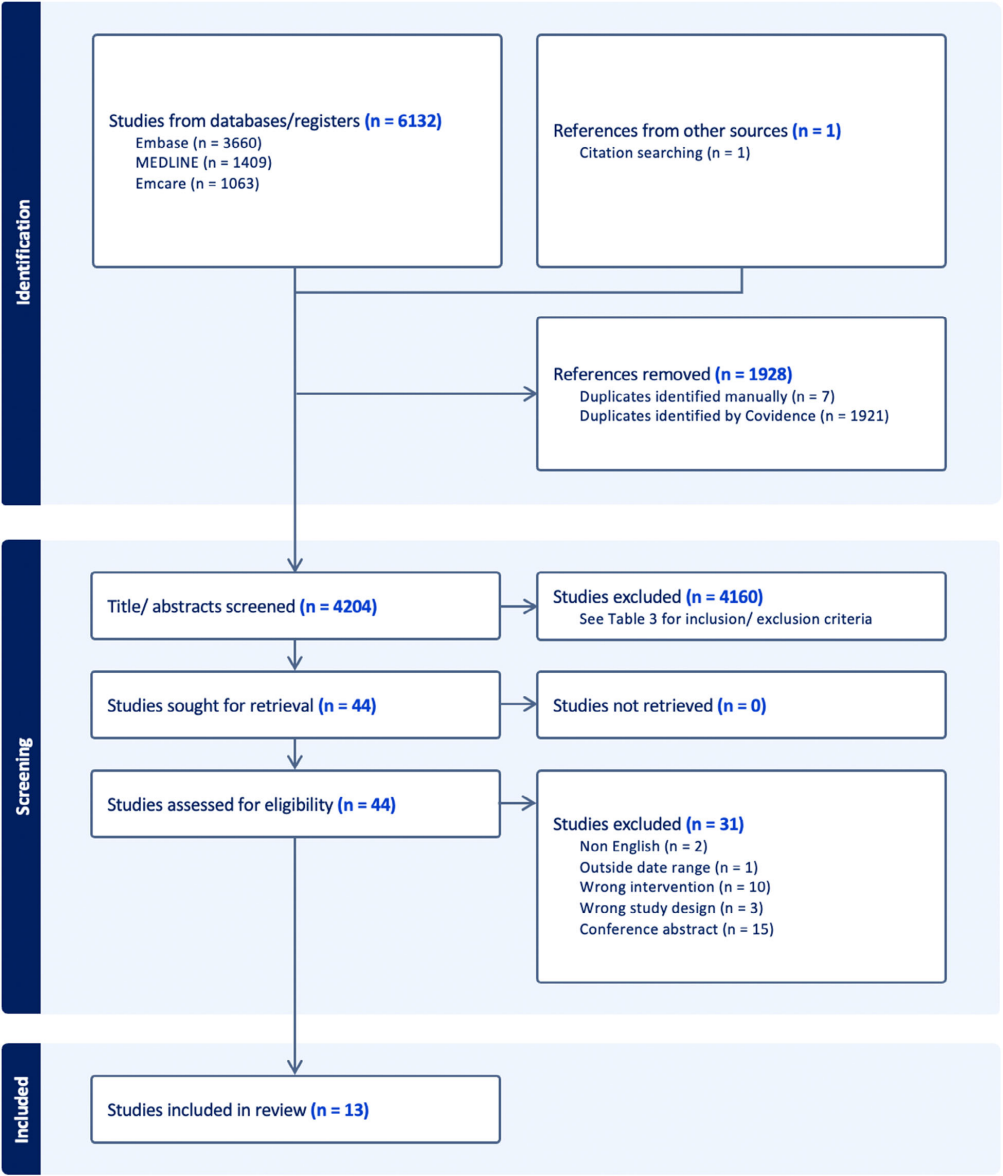
PRISMA flowchart for systematic review.

The literature search was conducted on 28 March 2023. The PRISMA flowchart (Fig. 1) demonstrates that 6132 articles were retrieved from the database search, and one was retrieved through citation searching. 4205 articles underwent title/abstract screening after duplicates were removed and 44 articles were included for full‐text review. 13 articles were included after full‐text review, with majority of articles excluded due to being conference abstracts (*n* = 15) or wrong intervention (*n* = 10).

Table 4 summarises the 13 articles reviewed, including the mode of translation used and CASP rating. Twelve articles were qualitative studies and one article was a cohort study. Only two studies were set in MI departments, with one explicitly exploring how translated recordings can improve image quality and reduce radiation exposure.^24^, ^25^ Five articles explored the use of MTAs with pre‐loaded phrases and five articles explored multiple modes of MTAs, including free‐text input and pre‐loaded phrases. Further, five articles were set in Australian public hospitals. All 13 articles were published in the English language.

## Discussion

### Benefits and enablers of MTAs

#### Availability

Mobile translation applications may offer a readily available mode of translation that is beneficial in out of hours care. MTAs can be accessed readily on portable devices, such as mobile phones or tablets, that can be brought directly to the point of care.^6^, ^8^, ^9^, ^10^, ^11^, ^12^, ^13^, ^14^, ^25^ Accessibility of the applications means that translation can be brought into MI examinations without delay, ensuring timely translation. It has also been suggested that MTAs are useful for daily conversations surrounding care where phoning an interpreter is excessive or when timely translation is needed.^6^, ^12^ Such interactions may include asking whether the patient has pain or needs to use the toilet. HCWs have also suggested that it is difficult to source professional interpreters after hours, and in turn delays necessary patient care.^6^ MTAs can be accessed on portable devices at any time of the day, negating the difficulty of accessing translation services out of hours. This is especially beneficial in the context of MI as many examinations occur after hours and are time sensitive.

#### Safety benefits

Mobile translation applications have the potential to improve patient and HCW safety through enhanced communication. Importantly, MTAs may also highlight potential risks to safety before they happen. Falls can occur in the MI department for a variety of reasons, including lower limb injuries, a previous history of falls, altered mental state and age.^26^ MTAs with pre‐loaded phrases have been used to educate patients about safety precautions such as being a falls risk.^9^ An MTA with this capability may in turn reduce the number of falls that occur in the MI department, but furthermore enhance imaging examinations by modifying imaging technique to best suit patient mobility. The ability for MTAs to ask targeted questions has also enhanced the quality of health assessments performed by HCWs in other healthcare disciplines.^9^, ^12^, ^25^ It is imperative to ascertain the three C's before commencing any MI examination, yet doing this accurately is difficult with patients with LEP. Indeed, failure to do so may lead to radiation safety incidents due to performing incorrect imaging for a CALD patient. Using MTAs in the absence of a professional interpreter may aid MI professionals to ask targeted questions in a way that CALD patients can accurately respond to, conducting a health assessment that MI professionals can confidently use to perform correct imaging and reduce radiation safety incidents.

Furthermore, enhanced communication through MTA use may lead to reduced radiation exposure for CALD patients due to enhanced image quality.^24^, ^25^ One study investigating the use of pre‐recorded breathing instructions for chest X‐rays of CALD patients reported a significant reduction in rejected chest X‐rays due to poor inspiratory effort following implementation.^24^ This clearly demonstrates that when positioning and breathing instructions are delivered so that CALD patients can understand, the number of repeat images due to suboptimal image quality will reduce. Hence, additional radiation exposure due to a repeat image will also reduce.

One study also suggested that MTAs may be useful in identifying a deteriorating patient without delay in sourcing a professional interpreter.^6^ Nursing staff found that an MTA with pre‐loaded phrases was beneficial in asking quick questions about the patient's condition and symptoms when they are deteriorating, meaning that there is minimal delay to intervention.^6^ It is a registration requirement that Australian medical radiation practitioners can recognise and act upon a deteriorating patient under the Medical Radiation Practice board of Australia (MRPBA) Professional Capabilities for Medical Radiation Practitioners.^27^ Thus, an MTA is a tool that MI professionals may be able to use to do so with CALD patients.

#### Enhance communication

The most notable benefit for MTAs is that they may improve the quality of communication between MI professionals and CALD patients. The inability to effectively communicate with CALD patients is a source of frustration for HCWs and can lead to poor bedside manner.^6^, ^10^, ^12^, ^15^ The purpose of MTAs is to overcome language barriers to enhance communication between CALD patients and HCWs and to better enable care. In one Australian hospital ward, nurses self‐reported that the use of MTAs with pre‐loaded phrases had improved the quality of communication with CALD patients, leading to a statistically significant reduction in nurse frustration when treating CALD patients (*P* = 0.008).^12^ Other studies have echoed this idea, suggesting that MTAs can be beneficial for mundane situations where sourcing a professional interpreter is not needed.^8^, ^9^, ^10^, ^12^ In MI, such situations may include asking if the patient feels pain, is nauseous or dizzy. An MTA that enables MI professionals to ask such questions may reduce the time taken to perform brief health assessments and ensure continued departmental efficiency.

Some studies have produced quantitative data surrounding how MTAs improve communication. The use of MTAs with pre‐loaded phrases has been investigated to perform allied health assessments.^9^, ^12^ Notably, one study reported that the time taken to perform the allied health assessment fell from 42 min to 15.6 min, and the allied health clinicians' ‘complete confidence’ in the quality of the assessment rose from 10% to 42%.^9^ This shows that clinicians had increased confidence that the CALD patient was able to understand during their interaction, which could also translate into MI. An MTA that is designed to incorporate written and recorded phrases may aid MI professionals with giving positioning instructions effectively and ensure that the patient is more informed about what is happening during their imaging examination.

It is worth noting that very few articles included in this review investigated patient experiences using MTAs. Limited research in this area may be attributed paradoxically to a language barrier and limited availability of professional interpreters. Patients have recounted difficulties experienced at understanding their nurses and adequately expressing their needs.^12^ After engaging with an MTA, one cohort of patients (*n* = 7) felt that communication with their nurse had enhanced and they were able to express their needs more easily, with six patients agreeing that the app was useful.^12^ Additionally, patients have been appreciative of the attempt to communicate their native language.^10^ Imaging examinations may be confronting for some patients considering the need for large equipment, positioning, and palpation. Thus, an MTA shows benefit in raising understanding and comfort surrounding their examination.

Furthermore, included articles investigating MTAs with pre‐loaded phrases did not include a full set of phrases used in the app. Although some of these articles provided examples of phrases used, it is not possible to say whether these available MTAs already have the necessary vocab to fully enhance communication for MI purposes. This is unlike free‐input MTAs, where the user would theoretically be able to input any MI related phrase and have it translated into the desired language.

### Limitations and barriers for MTAs

#### Reliability and accuracy

MTAs that require free input of speech or text can have large inaccuracies and can be a source of frustration and safety problems. In MI examinations, there may be higher‐risk conversations involved which include consenting to an invasive procedure, asking about medical history, allergies or medications. The question hence needs to be raised; can an MTA be relied on to make precise translations that uphold patient safety and care during an imaging examination? The possibility for severe translation errors in MTAs with free‐text and speech input is a common theme in the literature, especially as sentence complexity increases.^8^, ^10^, ^11^, ^16^, ^25^ One study noted nursing staff frustration and confusion at the failure of Google Translate™ to accurately translate questions that had been typed, leading to unsuccessful communication encounters between LEP patients and nurses.^10^ Furthermore, translation errors may not be culturally appropriate, and indeed could be offensive towards the patient.

The accuracy of Google Translate™ has been tested by translating a set of 100 patient care instructions from English to Spanish, and then back translating to English.^16^ It was found that nine Spanish translations were unsafe and did not reflect the original meaning of the patient care instructions, with more complex instructions being more prone to errors.^16^ Additionally, a good English to Spanish translation did not assure a good back translation into English (*R* ^2^ = 0.355), meaning that back translating into English is not a good measure of the accuracy of Google Translate™.^16^

Translation inaccuracies were only a flagged limitation for free‐text or speech input MTAs. MTAs with pre‐loaded phrases had been verified by professional translators and native speakers, and hence showed no inaccuracies.^8^, ^9^, ^10^ If inaccuracies are a concern for patient safety and care in the MI department, MTAs with pre‐loaded phrases are a possible solution. However, the one‐directional nature of communicating using MTAs with pre‐loaded phrases means that it may be difficult to confirm understanding with the CALD patient. In the context of high‐risk MI examinations, such as MRI safety screening, the risk of translation errors and miscommunication using MTAs outweighs their benefits due to the potential for severe safety repercussions.

#### Privacy and confidentiality

Mobile translation applications may present issues regarding confidentiality and data security for CALD patients in MI. A confidentiality issue presents when data can be saved about an individual.^13^ In the context of MI, information relating to symptoms, medical history and pregnancy status is sensitive information that is private. Hence, it is an important design feature of the MTA that it upholds confidentiality by not identifying patients or the location of their care, does not store data and uses a secure internet connection.^13^ The device that the MTA is installed on may also pose a risk to privacy and confidentiality.^10^, ^13^ The use of personal devices has been flagged as an ethical and privacy issue, and it is often against organisational policy to use personal devices when interacting with patients.^10^, ^13^ It is for this reason that many studies included in this review used MTAs installed on devices approved and provided by the organisation, such as iPads which were to remain in the workplace. It would be suggested that MTAs used in MI would also be installed on organisation‐approved devices that remain in the department after use to uphold patient privacy in accordance with departmental policy.

#### Use in practice

While MTAs are suggested to enhance communication, the question needs to be raised whether they are practical to use in MI. HCWs using the apps have reported that MTAs need to be installed on portable devices that are close to the point of care so that they are more likely to reach for the device and incorporate it's use into routine care with CALD patients.^6^, ^8^, ^12^ Further, infection control has been flagged as an important consideration due to the cross‐infection risk of HCWs treating multiple patients consecutively.^9^, ^11^ In MI, a safe example of device integration could be placing an iPad with a protective case on charge in the X‐ray room next to the console so that it is easy to retrieve and is safe to wipe with an appropriate disinfectant.

Some HCWs found it difficult to include MTAs in their routine when treating CALD patients.^9^ When HCWs did not use MTAs provided to them in three Australian aged care wards, 66% of HCWs said this was due to a translating family member being present, which is not best practice due to personal bias and/or poor language skills.^6^, ^10^ Further, 33% were due to time pressures and 33% as it was easier to ‘get by’ without it.^10^ Overall, this suggests that MTAs may be an aid tool that is not always necessary to deliver effective patient care.

Furthermore, it has been reported that MTAs may not be beneficial for CALD patients who speak a unique dialect, have low technological literacy, cannot read, or have cognitive impairment.^6^, ^8^, ^12^, ^13^, ^25^ In these cases, patients may find the MTAs difficult to understand, leading to increased confusion surrounding their care. Studies have noted the importance of using devices with large screens and audiovisual cues, such as audio recordings and pictures, to aid translation for patients with low literacy and mental impairment.^9^, ^10^ It is important to consider device size, font size and multi‐modal translation with audiovisual elements when designing MTAs for use in MI in order to create an inclusive application that can be used for many CALD patients. Additional dialects should also be considered and included.

The one‐directional nature of conversations in MTAs with pre‐loaded phrases has also been flagged as a limitation as it does not allow the CALD patient to effectively give detailed information or follow‐up questions in response to their HCW.^10^, ^11^, ^13^ These MTAs only allow for simple responses from a set list of pre‐translated phrases.^10^, ^11^, ^13^ As a result, HCW's may be able to ask questions that their CALD patients understand, but not receive clear answers in return. In the context of MI, this may only be a problem when the questions being asked by MI professionals are more complex or open‐ended. Simpler, closed‐ended questions may be used to effectively garner information from CALD patients. Further, the inability of the CALD patient to give a detailed reply may not be a limitation of free‐text input or speech‐to‐speech MTAs. Using this mode, CALD patients can type or say their response or follow‐up question in their preferred language for this to be translated into the preferred language of the HCW.

Importantly, MTA developers need to consider whether their MI organisation permits machine translation. Machine translation is forbidden in five hospitals in the United States with paediatric services due to inaccuracy and safety concerns.^28^ Using or developing an MTA is futile if the technology is not permitted.

### Limitations

This systematic review was made rigorous through a comprehensive search strategy and article screening process. Some limitations are recognised, however. Firstly, only two MI‐related papers were included in the review, with only one focusing on outcomes from the introduction of pre‐recorded translations in an X‐ray department. With a limited investigation of translation in one imaging modality, the transferability of the results of these studies into other imaging modalities, such as CT or MRI, may be limited. Most papers included focused on the use of MTAs by nursing, medical and allied health staff who may have different requirements for an MTA compared to MI professionals such as radiographers, sonographers and nuclear medicine technologists. Hence, findings relating to the usefulness and effectiveness of MTAs may not transfer wholly to MI applications. Included studies also mostly reported using qualitative data from surveys or focus groups. Bias may be present as subjects were self‐reporting about their experiences using MTAs. Lastly, the perspectives of CALD patients towards MTAs were not explored in detail. We suggest further research to ascertain whether CALD patients find MTAs useful for communicating during healthcare interactions and to have their input for important design features.

## Conclusion

Findings from this systematic review indicate that MTAs are accessible and may enhance communication and safety of CALD patients and MI professionals in Australia. By theoretically applying the literature to the Australian setting, it was found that different modes of MTAs show benefits and limitations with regards to their accuracy and usability, however, MTAs with pre‐loaded phrases show greatest accuracy and safety when communicating in low‐risk conversations. When designing MTAs for Australian MI departments, it is recommended that a variety of useful phrases be included with audiovisual cues and a variety of ways for CALD patients to respond. MTAs should be installed on devices close to the point of care and be large enough for patients to view. Importantly, we recognise that professional interpreters remain the gold standard for medical translation, especially during complex MI examinations. However, MTAs may present a viable alternative for low‐risk conversations in MI when professional interpreters are absent and could in turn alleviate pressures on translation services.

## Conflict of Interest

The authors declare no conflict of interest.

## Supporting information


**Appendix S1.** PRISMA checklist.

**Appendix S2.** CASP checklist.

## Data Availability

Data sharing not applicable to this article as no datasets were generated or analysed during the current study.
