# Supplementary material for: Exploring the use of mobile translation applications for culturally and linguistically diverse patients during medical imaging examinations in Australia – a systematic review
Source: J Med Radiat Sci. 2024 Jan 22;71(3):432–44. doi: 10.1002/jmrs.755 (PMC11569402; doi:10.1002/jmrs.755)
Supplement: Supplementary file 2 — Appendix S2. CASP checklist. [file JMRS-71-432-s002.docx]

| CASP Qualitative Checklist | Albrecht et al.  2013 (8) | Choong et al. 2021 (20) | Davis et al.  2019 (23) | Day & Song 2017 (6) | Freyne et al.  2018 (9) | Freyne et al.  2015 (14) | Hwang et al.  2022 (10) | Miller et al.  2018 (16) | Pocock et al. 2020 (15) | Panayiotou et al. 2020 (11) | Panayiotou et al. 2019 (13) | van Vuuren, van Dyk & Mokoena 2021 (21) |
| --- | --- | --- | --- | --- | --- | --- | --- | --- | --- | --- | --- | --- |
| 1. Clear statement of aims? | 2 | 2 | 2 | 1 | 2 | 2 | 2 | 2 | 2 | 2 | 2 | 2 |
| 1. Appropriate qualitative method? | 2 | 2 | 2 | 2 | 2 | 2 | 2 | 2 | 2 | 2 | 2 | 2 |
| 1. Appropriate reseach design? | 2 | 2 | 2 | 2 | 2 | 1 | 2 | 2 | 2 | 2 | 2 | 1 |
| 1. Appropriate recruitment strategy? | 1 | 1 | 2 | 2 | 2 | 2 | 2 | 1 | 2 | 2 | 1 | 1 |
| 1. Appropriate data collection method? | 2 | 2 | 2 | 2 | 2 | 2 | 2 | 2 | 2 | 2 | 2 | 1 |
| 1. Relationship b/w researcher and participants? | 2 | 2 | 2 | 2 | 2 | 0 | 2 | 0 | 2 | 2 | 2 | 2 |
| 1. Ethical issues? | 2 | 0 | 2 | 2 | 2 | 0 | 2 | 0 | 2 | 2 | 2 | 2 |
| 1. Rigorous data analysis? | 2 | 0 | 2 | 2 | 2 | 1 | 2 | 2 | 2 | 2 | 2 | 2 |
| 1. Clear statement of findings? | 2 | 1 | 2 | 2 | 2 | 0 | 2 | 2 | 2 | 2 | 2 | 2 |
| 1. Valuable research? | 2 | 2 | 2 | 2 | 2 | 1 | 2 | 2 | 2 | 2 | 2 | 2 |
| Total /20 | 19 | 12 | 20 | 19 | 20 | 11 | 20 | 15 | 20 | 20 | 19 | 17 |
| % | 95 | 70 | 100 | 95 | 100 | 55 | 100 | 75 | 100 | 100 | 95 | 85 |
|  |  |  |  |  |  |  |  |  |  |  |  |  |
| Cohort Study CASP Checklist | Silvera-Tawil et al. 2021 (12) |  |  |  |  |  |  |  |  |  |  |  |
| 1. Study addressed the issue? | 2 |  |  |  |  |  |  |  |  |  |  |  |
| 1. Acceptably recruited cohort? | 2 |  |  |  |  |  |  |  |  |  |  |  |
| 1. Exposure accurately measured? | 2 |  |  |  |  |  |  |  |  |  |  |  |
| 1. Outcome accurately measured? | 2 |  |  |  |  |  |  |  |  |  |  |  |
| 1. Confounding factors identified? | 2 |  |  |  |  |  |  |  |  |  |  |  |
| 1. Confounding factors accounted for? | 1 |  |  |  |  |  |  |  |  |  |  |  |
| 1. Follow-up complete? | 2 |  |  |  |  |  |  |  |  |  |  |  |
| 1. Follow-up long enough? | 1 |  |  |  |  |  |  |  |  |  |  |  |
| 1. Results? | 2 |  |  |  |  |  |  |  |  |  |  |  |
| 1. Estimate of treatment effect? | 1 |  |  |  |  |  |  |  |  |  |  |  |
| 1. Results believeable? | 2 |  |  |  |  |  |  |  |  |  |  |  |
| 1. Results applicable? | 2 |  |  |  |  |  |  |  |  |  |  |  |
| 1. Results congruent with other evidence? | 2 |  |  |  |  |  |  |  |  |  |  |  |
| Total /25 | 21 |  |  |  |  |  |  |  |  |  |  |  |
| % | 84 |  |  |  |  |  |  |  |  |  |  |  |

Legend: 2 = yes; 1 = unclear; 0 = no
